# Modeling of Mechanical Properties of Threads Using Nonlinear Rheological Models

**DOI:** 10.3390/ma18091982

**Published:** 2025-04-27

**Authors:** Aleksandra Prążyńska, Zbigniew Mikołajczyk, Maciej Kuchar

**Affiliations:** 1 Faculty of Material Technologies and Textile Design, Textile Institute, Lodz University of Technology, 116 Zeromskiego Street, 90-924 Lodz, Poland; zbigniew.mikolajczyk@p.lodz.pl; 2 Department of Vehicles and Fundamentals of Machine Design, Faculty of Mechanical Engineering, Lodz University of Technology, 1/15 Stefanowskiego Street, 90-924 Lodz, Poland; maciej.kuchar@p.lodz.pl

**Keywords:** dynamic stretching tests, multi-filament synthetic threads, rheological models of textiles, numerical modelling

## Abstract

This publication presents the results of research aimed at analyzing the dynamic stretching process of multifilament polyester and polyamide threads with medium linear densities. Building virtual design tools for the mechanical properties of textiles under dynamic impact conditions on their structures is a fundamental challenge for identifying textile technological processes and their behavior in real operating conditions. In the Autodesk^®^ Inventor^®^ software environment, a virtual analogue model was built based on appropriately connected modules of the Kelvin–Voigt rheological models, for which the input parameters of the selected rheological models were defined. The nonlinear static and dynamic elasticity coefficients were determined based on the obtained results of experimental tests carried out in static conditions on a testing machine at a speed of 33 × 10^−6^ m/s and in dynamic conditions on a constructed measuring device. The nonlinear viscosity coefficient was calculated based on data read from the force-time characteristics obtained by measuring forces during stress relaxation in the threads. To conduct this research, an original research stand was designed and built. A series of simulation calculations of the dynamic stretching process were performed for different values of linear densities, lengths of stretched thread sections, and stretching speeds. A comparative analysis of the characteristics obtained from experimental and modeling studies was performed. A very good agreement between the experimental and numerical simulation curves was obtained, which leads to the conclusion about the usefulness of the tools used in the work for the physical description of the thread stretching process.

## 1. Introduction

The functional properties of textile materials are largely determined by the properties of the fibers from which these products are made. Due to the complexity of macroscopic, molecular, and supramolecular structure, the behavior of fibers under the influence of applied external forces is more complex than the behavior of many solids. The principles of viscoelastic body mechanics are used to describe the mechanical properties of fibers that depend on time. Fibers and yarns are characterized by a significant length in relation to their transverse dimensions, which is why most studies consider deformability caused by axial tensile forces [1].

To describe the theoretical behavior of threads and textiles under the influence of an applied tensile force, mechanical rheological models are used, which are a combination of a perfectly elastic body model (Hooke’s model), a viscous fluid model (Newton’s model), and a plastic body model (Saint Venant’s model). Simple two-parameter Maxwell and Kelvin–Voigt models are characterized by the ability to relax only or to creep only and therefore do not describe the actual behavior of viscoelastic bodies. To capture the phenomena occurring under the influence of applied tensile stresses, more complex three-, four-, and more parameter models are used, which include both those properties that allow for a more accurate representation of the behavior of a viscoelastic material.

As mechanical models are expanded, the order of state equations increases. This entails increasingly complicated solutions. Building complex mechanical models is not particularly difficult; the problems begin only when solving the corresponding state equations and determining the values of individual parameters.

The above issues, important in the context of analyzing the mechanical properties of textiles, prompted the authors to articulate the main research objective, which is to develop a validated model for identifying the behavior of textile materials in real conditions of manufacturing processes and their usage.

## 2. Literature Review

Yarns can be classified as solids with highly complex mechanical properties. Under the influence of applied external forces, the behavior of yarns is significantly more complex than that of many solids. Due to the characteristic time-dependent properties of yarns, the use of viscoelastic body mechanics principles will facilitate the description of these phenomena [1].

A body subjected to a system of forces deforms, experiencing both relative elongations and shape deformations [2]. In the case of bodies with a length significantly greater than their transverse dimensions, such as fibers or yarns, loaded in the longitudinal direction, the relationship between stress σ, strain ε, and time t is considered. The rheological equation of state (RES) for one-dimensional bodies describes the dependence:

(1) $$R_{i}\;(\sigma ,\;\varepsilon ,\;t)=0\;\;\mathrm{for}\;i=1,\;2,\;\ldots \;n$$

where σ—stress components; ε—strain components; n—number of state equations.

To describe the behavior of threads and textiles subjected to tensile loads, a number of rheological models have been developed. The rheological properties of textile materials (fibers, yarns, knits, fabrics, and nonwovens) are most often modeled using well-known mechanical systems with two or three parameters and complete systems that combine Hookean bodies and Newtonian dampers. More complex models are also introduced, taking into account nonlinear elastic bodies and friction elements with constant and nonlinear dry friction.

The Kelvin–Voigt model, connected in series with a spring or dashpot, and the Eyring model were used to describe the creep process of warp knitted fabrics. Among the selected viscoelastic models, the best fit of the model characteristics to the experimental ones was obtained for the Kelvin–Voigt model connected with a dashpot [3].

The analysis of stress relaxation in knitted fabrics made of shape-memory polymers (SMP) showed that the process is best described by the Kelvin–Voigt model combined in parallel with the Maxwell model [4].

According to Szosland and Czołczyński [5], most textile raw materials can be classified as bodies in which stress and strain relaxation occur under the influence of a suddenly applied load. These properties, according to the authors, can be represented using the linear Zener model. B. Włodarczyk and K. Kowalski [6] described the influence of yarn heterogeneity on the forces occurring during the pulling of threads through a friction barrier using the Zener model. The fractional Zener model and the extended Lomintz model were used to describe the viscoelastic properties of textile composites [7].

In the article [8], the authors analyzed the application of rheological models that would most accurately characterize the elastic properties of polypropylene yarn. To determine the effect of dyeing the fibers in the solution on their viscoelastic properties, they used the Vangheluwe model and the Zurek model and their modifications. The best model for describing the shape of the stress–strain curve for black-dyed fibers is the modified Vangheluwe model, while the Zurek model best reflects the stress–strain characteristics obtained for raw and blue-dyed fibers.

In publication [9], the authors analyzed the Vangheluwe, Zurek, and Manich rheological models in the MATLAB^®^ 7.6 environment to assess their suitability for describing the behavior of cotton yarns with added elastane subjected to tensile tests using a Lloyd LR5K dynamometer (AMETEK STC, Berwyn, PA, USA). The initial parameters for each predictive model were the strength and elongation at break. Through tensile tests conducted on some core yarns with added elastane, they determined the precise coefficients of the nonlinear equations of the rheological models. All three models selected for analysis accurately reflect the behavior of the tested yarns subjected to the stretching process. As a result of their work, the authors developed a tool for optimizing the composition and characteristics of the produced yarns.

In article [10], the authors applied a modified Zurek model to describe the relaxation of polypropylene monofilament. The proposed model was verified using three types of polypropylene monofilaments with different diameters: 0.15, 0.30, and 0.45 mm. The loading and relaxation phases were obtained using an Instron 4204 tensile testing machine (Instron, Norwood, MA, USA). Each type of monofilament was subjected to a tensile test at speeds of 5, 50, and 500 mm/min up to an elongation of 15%. The samples were then subjected to relaxation for 180 s. Based on the conducted studies, it was found that the proposed model describes the relationships between the stress and strain of polypropylene monofilaments subjected to tensile testing and the relaxation phenomenon of the tested materials.

H. Helali, A. Babay, and S. Msahli [11] investigated the nonlinear behavior of core elastic yarns based on the development of the Vangheluwe model, which is a parallel connection of two Maxwell elements and a nonlinear source of coefficient C. It was found that the selected model provides the best response to the nonlinear viscoelastic behavior of the tested yarns and provides a better fit to the data obtained during the experimental studies.

In article [12], the authors conducted dynamic tensile, compression, and shear strength tests on woven and knitted polypropylene thermoplastic composites reinforced with glass fibers. Measurements were carried out in the range of 10^−5^ to 10^3^ 1/s. The Burgers rheological model was used to represent the deformation characteristics of the tested samples, while the Zener model described the brittleness of the glass fibers under the applied stresses. The conducted research led to the development of an effective method for identifying parameters necessary for numerical analysis of the mechanical properties of composites.

J. Yekrang and D. Semnani investigated the mechanical properties in the longitudinal and transverse directions of cylindrical weft knitted fabrics used in medical and tissue engineering [13]. The measurements were carried out on a testing machine at a speed of 10 mm/min for twenty variants of structures in the wet state in order to reproduce the conditions of use. To describe the mechanical properties of tubular knitted fabrics during unidirectional stretching, they selected seven different rheological models: the Maxwell model, the Maxwell model with a nonlinear spring, the Kelvin model, the Kelvin model with a nonlinear spring, the Zener model, the Vangheluwe model, the Kelvin model with a nonlinear spring (according to the power rule). They described each of the models with an equation, based on which they calculated the rheological coefficients of the individual elements. Comparison of the experimental and theoretical results showed that the stretching process is best described by the Kelvin model with a nonlinear spring.

In the articles [14,15], a new model of the behavior of natural yarn (cotton), artificial yarn made of natural polymers (Tencel), and synthetic yarn (polyester) during stretching was presented based on the mathematical description of the stress–strain characteristic. Due to the variable course of the stretching characteristics, they were divided into three parts, and each of them was described by an appropriate rheological model, i.e., the Vangheluwe and Zurek model and their modifications and the Legrand model. In the first stage, the parameters of the individual models were determined in the MATLAB environment, and their influence on the shape of the stretching curve was analyzed. Comparison of the obtained model characteristics with the experimental ones showed that the proposed approach to the problem allowed for a correct but not ideal representation of the stretching process in the case of most of the yarns used.

In the publication [16], fatigue tests of cotton plain jersey fabric simulating conditions occurring during the production process and use of knitted clothing were presented. The tests of structure deformations under the influence of cyclic loading were carried out using a designed device allowing the displacement of the movable clamp in the range of 40 to 80 mm in the longitudinal direction for a different number of cycles. After the measurement, the samples were subjected to a relaxation process, periodically measuring their length for 500 h. The authors concluded that the model that best reproduces the viscoelastic properties of the tested samples is the Burgers model.

In article [17], a method for determining the allowable load of SSB (Sheet Support Binder) fabric at the joints was presented. Tensile strength tests along the weft of 40 mm wide samples were conducted on a Zwick testing machine (Zwick/Roell, Ulm, Germany). The distance between the clamps was 200 mm, and the measurement speed was 80 mm/min. Based on the obtained experimental results, a simulation of the behavior of SSB fabrics in the joint area during operation was carried out. To describe the phenomena occurring due to the tensile force, the Leserič rheological model, which is a series connection of the Newton model and the Kelvin model, was adopted. The analysis of the results allowed the authors to conclude that the selected model correctly describes the behavior of the material up to the creep limit of the fabric.

The article [18] presented the studies of the evaluation of material properties of thermoplastic polymers (PLLA) used for the production of stents. The strength tests were carried out on a Zwick tensile tester with the stretching speed of 2 and 5 mm/min. The PRF rheological model was selected to represent the viscoelastic behavior of the applied material and implemented in the ABAQUS 2013 version (6.13) program. The simulation using the finite element method allowed for the identification and optimization of the parameters of thermoplastic polymers subjected to large deformations.

The publication [19] presented the results of experimental and analytical tests of the t-ECCY composite yarn. Samples of 500 mm length were subjected to a stretching process at a speed of 100 mm/min at different strain values (0.1, 1, and 2/mm). To represent the elastic properties of the composite yarn, the authors chose the Vangheluwe model connected in series with the Hooke model. The calculated correlation coefficient comparing the experimental data with the theoretical data was R = 0.999, which indicates a good fit of the proposed model.

The article [20] presents the research on the properties of core polyurethane yarns. Samples of 500 mm length were subjected to stretching at a speed of 500 mm/min on the USTER TENSORPID III testing machine (Uster Technologies AG, Uster, Switzerland). To analyze the properties of the tested yarns, a nonlinear model consisting of a Maxwell model connected in series with a nonlinear and linear spring was proposed. Based on theoretical calculations and experimental results, it was found that it is possible to use the selected model to characterize the behavior of core polyurethane yarns during the stretching process.

The publication [21] presented stress relaxation tests of VORTEX yarns with linear densities of 8.2 tex and 32.4 tex produced on a Murata spinning machine (Murata Machinery, Ltd., Kioto, Japan) from bamboo and cotton fibers in the proportion of 70/30%. To determine the effect of yarn thickness on relaxation properties, the threads were subjected to a stretching process to 4% elongation at a speed of 200 mm/min. To assess the effect of tensile stress and stretching speed, stress relaxation tests lasting 300 s were carried out with tensile elongation in the first case from 3 to 5% and in the second up to 4%. In addition, to examine the effect of stretching speed on relaxation properties, measurements were taken for three yarn stretching speeds of 50, 200, and 350 mm/min. To predict and analyze the stress relaxation properties of VORTEX yarns, the author used a modified, generalized Maxwell model. The obtained research results showed that the applied rheological model can be used to describe the stress relaxation mechanism of the tested yarns.

## 3. Numerical Simulation of the Dynamic Thread Stretching Process for Nonlinear Input Parameters of Rheological Models

Simulation is an approximate reproduction of physical phenomena, often implementing their mathematical model. Simulation techniques are used primarily in situations where determining the solution by analytical method is not possible or very laborious. Computer simulation is a tool that allows predicting the behavior of bodies and objects during their use. It is currently used in all fields of science and technology. It allows reducing the number of prototypes produced, shortening the time of tests conducted on them, and thus increasing the efficiency and reducing the costs of production processes [22,23].

The experimental study of phenomena occurring during dynamic stretching of threads is a complex process requiring numerous measurements and the use of specialist devices. The possibility of using the numerical simulation method instead of bench tests is faster and cheaper, of course, provided that reliable results are obtained. Hence, there is a need to verify the results obtained from virtual dynamic models in real conditions.

The simulation of the dynamic thread stretching process was carried out based on the experimental results presented in the article “Experimental Identification of the Mechanical Properties of Polymer Threads under Extreme Stretching Process Conditions” in the context of comparing the obtained results with theoretical data.

Dynamic thread stretching tests were performed on an original device designed and built according to the author’s concept, and a methodology for measuring and recording test results was developed. The measurement results allow for the correct mapping of the characteristics of the thread stretching process at high speeds. The measurements were performed for polyamide threads with a linear density of 56 and 156 dtex and polyester threads with a linear density of 55, 111, and 167 dtex. These threads constitute an important assortment group of raw materials used in knitting technologies for clothing and technical products, including bulletproof vests, impact barrier nets, aircraft composites, parachute canopy lines and threads, specialist products for extreme sports, textile composites in the automotive industry, etc. Thread sections with a length of l = 200 ÷ 1000 mm were subjected to stretching at speeds in the range of V = 10 ÷ 100 m/s. The adopted speeds correspond to the speeds occurring during the manufacturing and use of textile materials. The lengths of the stretched sections correspond to the measured sections of the threads in the feed zone and between the working elements on the knitting machines. The total number of measurement variants was 150.

**Figure 1:**
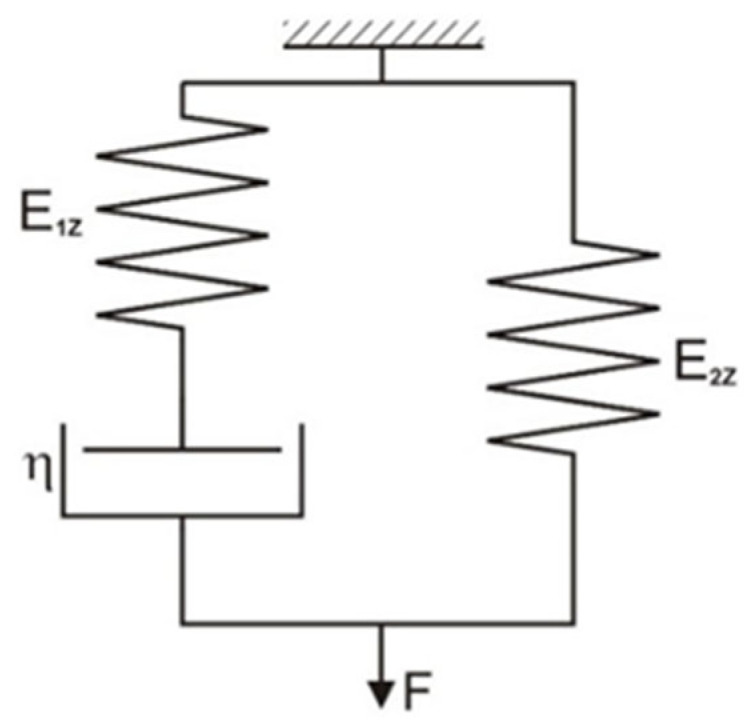
Zener model.

**Figure 2:**
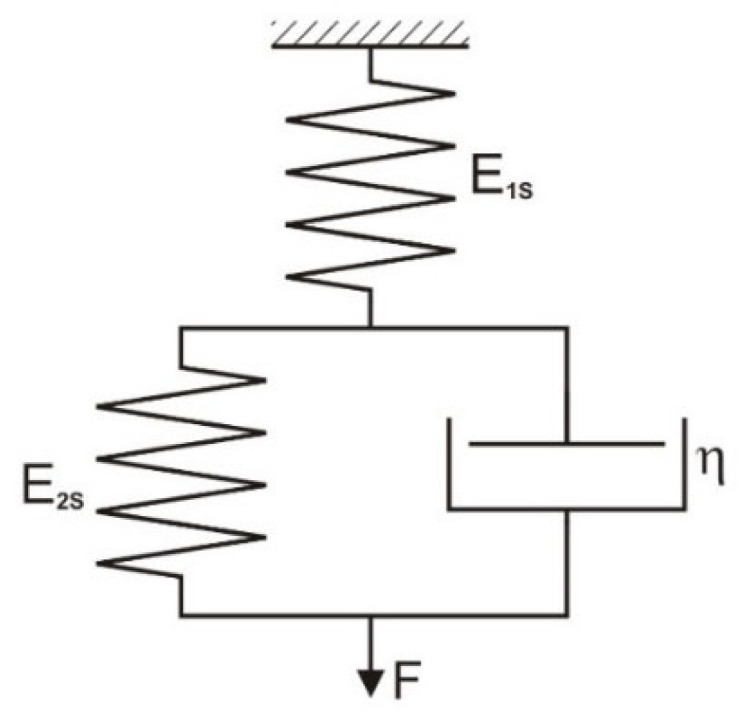
Standard 2 model.

Simulation studies of the dynamic thread stretching process were based on two three-parameter rheological models, i.e., the Zener model (Figure 1) and the Standard 2 model (Figure 2), which describe both the creep and relaxation processes. They are often used in textiles to model the behavior of viscoelastic bodies. The advantage of these models is the possibility of describing them using simple mathematical relationships, determining parameters experimentally, and using them in numerical methods [24,25].

In this publication, unlike most models described in the literature with linear parameters, research was undertaken on nonlinear models for which the following input parameters were defined: nonlinear coefficients of static and dynamic elasticity and nonlinear coefficient of viscosity.

### 3.1. Constitutive Relationships of Selected Rheological Models

Characteristics of the standard three-parameter model 1 (Zener) (Figure 1).

Equation of state:

(2) $$\frac{dƐ}{dt}\cdot \left(E_{1Z}+E_{2Z}\right)\cdot \eta +\varepsilon \cdot E_{1Z}\cdot E_{2Z}=\eta \cdot \frac{dF}{dt}+E_{1Z}\cdot F$$

Solution of the state equation for the initial condition t = 0, F(0) = F_0_:

(3) $$F\left(t\right)=F_{0}\cdot exp\left(-\frac{E_{1Z}\cdot t}{\eta }\right)+\eta \cdot \frac{d\varepsilon }{dt}\cdot \left(1-exp\left(-\frac{E_{1Z}\cdot t}{\eta }\right)\right)+E_{2Z}\cdot \varepsilon$$

for $\frac{d\varepsilon }{dt}=\omega =const$.

(4) $$F\left(t\right)=F_{0}\cdot exp\left(-\frac{E_{1Z}\cdot t}{\eta }\right)+\eta \cdot \omega \cdot \left(1-exp\left(-\frac{E_{1Z}\cdot t}{\eta }\right)\right)+E_{2Z}\cdot \omega \cdot t$$

The elasticity coefficients are related by the following equations:

*E*_1*Z*_ = *E_dZ_* − *E*_2*Z*_ (5)

*E*_2*Z*_ = *E_K_* (6)

*E*_1*Z*_ = *E_dZ_* − *E_K_* (7)

where E_1Z_, E_2Z_—elasticity coefficient for the Standard Model 1 (Zener); ω = dε/dt—rate of relative elongation increase; η—viscosity coefficient; ε—relative elongation; F—force; F_0_ –initial tension; T—stretching time; E_dZ_—elasticity coefficient determined for the Zener model under dynamic thread loads; E_K_—static elasticity coefficient.

Characteristics of the standard three-parameter model 2 (Standard 2) (Figure 2).

Equation of state:

(8) $$\left(E_{1S}+E_{2S}\right)\cdot F+\eta \cdot \frac{dF}{dt}=E_{1S}\cdot E_{2S}\cdot \varepsilon +\eta \cdot E_{1S}\cdot \frac{d\varepsilon }{dt}$$

The general form of the equation of state:

(9) $$a_{1}\cdot F+a_{2}\cdot \frac{dF}{dt}=b_{1}\cdot \varepsilon +b_{2}\cdot \frac{d\varepsilon }{dt}$$

where $a_{1}=E_{1S}+E_{2S}$; $a_{2}=\eta$; $b_{1}=E_{1S}\cdot E_{2S}$; $b_{2}=\eta \cdot E_{1S}$.

The solution to the equation for t = 0, F(0) = F_0_ is as follows:

(10) $$F\left(t\right)=F_{0}\cdot exp\left(-\frac{a_{1}}{a_{2}}\cdot t\right)+\frac{d\varepsilon }{dt}\cdot \frac{\left(-b_{1}\cdot a_{2}+b_{1}\cdot a_{1}\cdot t+b_{2}\cdot a_{1}\right)}{{a_{1}}^{2}}-exp\left(-\frac{a_{1}}{a_{2}}\cdot t\right)\cdot \frac{d\varepsilon }{dt}\cdot \frac{-b_{1}\cdot a_{2}+b_{2}\cdot a_{1}}{{a_{1}}^{2}}$$

for $\frac{d\varepsilon }{dt}=\omega =const.$

(11) $$F\left(t\right)=F_{0}\cdot exp\left(-\frac{a_{1}}{a_{2}}\cdot t\right)+\omega \cdot \frac{-b_{1}\cdot a_{2}+b_{1}\cdot a_{1}\cdot t+a_{1}\cdot b_{2}+exp\left(-\frac{a_{1}}{a_{2}}\cdot t\right)\cdot b_{1}\cdot a_{2}-exp\left(-\frac{a_{1}}{a_{2}}\cdot t\right)\cdot b_{2}\cdot a_{1}}{{a_{1}}^{2}}$$

The elasticity coefficients E_1S_, E_2S_, and E_K_ are related by the following equations:

(12) $$\frac{1}{E_{K}}=\frac{1}{E_{1S}}+\frac{1}{E_{2S}}$$

*E*_1*S*_ = *E_dS_* (13)

Hence,

(14) $$E_{2S}=\frac{E_{K}\cdot E_{1S}}{E_{1S}-E_{K}}\;\mathrm{or}\;E_{2S}=\frac{E_{K}\cdot E_{dS}}{E_{dS}-E_{K}}$$

where E_1S_, E_2S_—elasticity coefficient for Standard 2 model; ω = dε/dt—rate of relative elongation increase; η—viscosity coefficient; ε—relative elongation; F—force; F_0_—initial tension; T—stretching time; $a_{1},\;a_{2},\;b_{1},\;b_{2}$—coefficients in the Standard 2 model equation; E_dS_—elasticity coefficient determined for the Standard 2 model under dynamic thread loads; E_K_—static elasticity coefficient.

### 3.2. Determination of Nonlinear Parameters of Rheological Models, Elasticity Coefficients, and Viscosity Coefficient

#### 3.2.1. Determination of the Nonlinear Modulus of Elasticity Ed Under Dynamic Thread Loads

**Figure 3:**
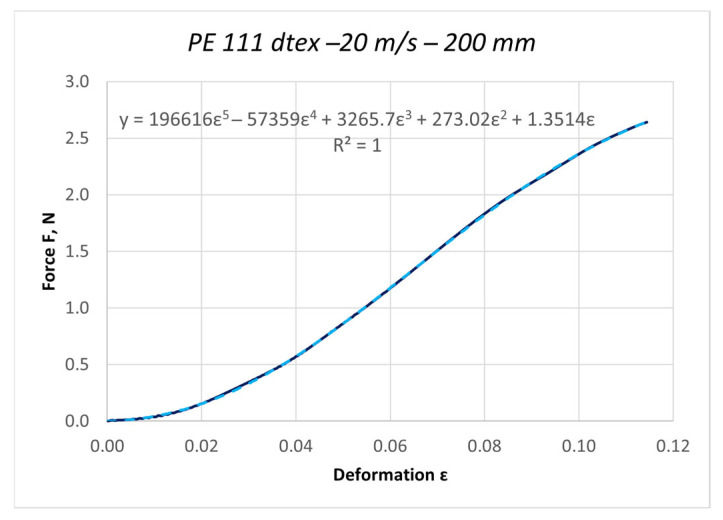
Approximation of the characteristic F = f(ε) with a fifth-degree polynomial for the PES 111 dtex polyester thread in the dynamic stretching process at a speed of 20 m/s.

**Figure 4:**
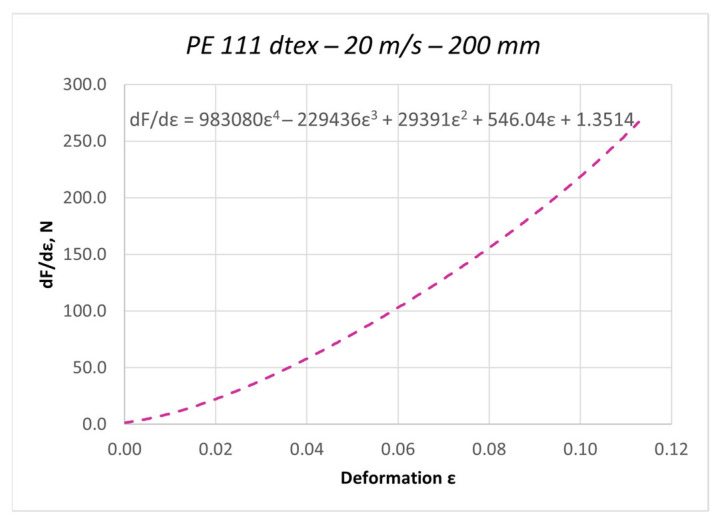
The course of the first derivative of the approximated characteristic F = f(ε) for the PES 111 dtex polyester thread in the process of dynamic stretching at a speed of 20 m/s (nonlinear modulus of elasticity).

The curves obtained in the dynamic stretching process, presented in the form of force characteristics as a function of deformation, were approximated by a fifth-degree polynomial. The values of the elasticity coefficient Ed were determined by calculating the first derivative of the function F = f(ε). Based on the determined curves, nonlinear changes in the dynamic elasticity coefficient of individual threads as a function of deformation can be determined. The obtained results of approximation and the first derivative are presented as an example for the PES 111 dtex polyester thread in Figure 3 and Figure 4.

#### 3.2.2. Determination of the Nonlinear Static Elasticity Coefficient E_K_

To determine the nonlinear characteristics of the elasticity coefficient E_K_, the 500 mm long threads used in the tests were subjected to a stretching process on a testing machine at a speed of 33 × 10^−6^ m/s. The curves obtained in the stretching process were presented in the form of force characteristics as a function of deformation F = f(ε) and approximated by a fifth-degree polynomial. As a result of the approximation and calculation of the first derivative with respect to the variable ε for each of the tested threads, an equation was obtained that allows for determining the nonlinear change of the elasticity coefficient E_K_.

**Figure 5:**
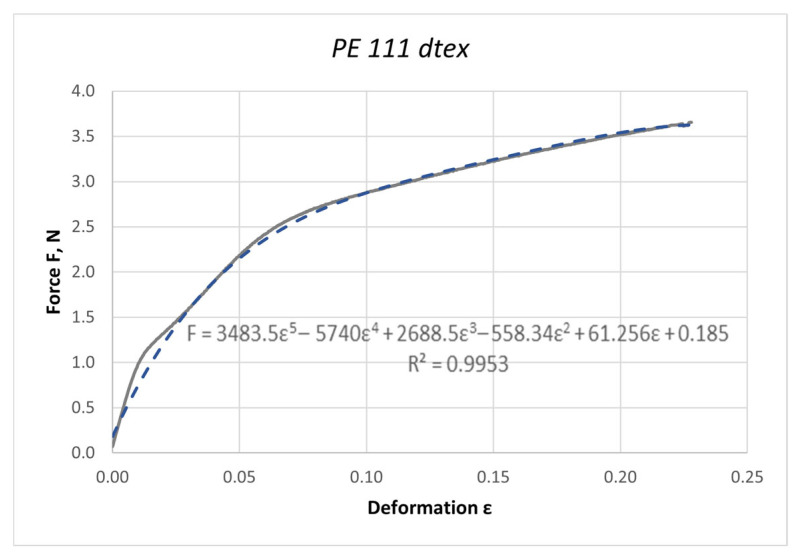
Approximation of the characteristic F = f(ε) with a fifth-degree polynomial for the PES 111 dtex polyester thread.

**Figure 6:**
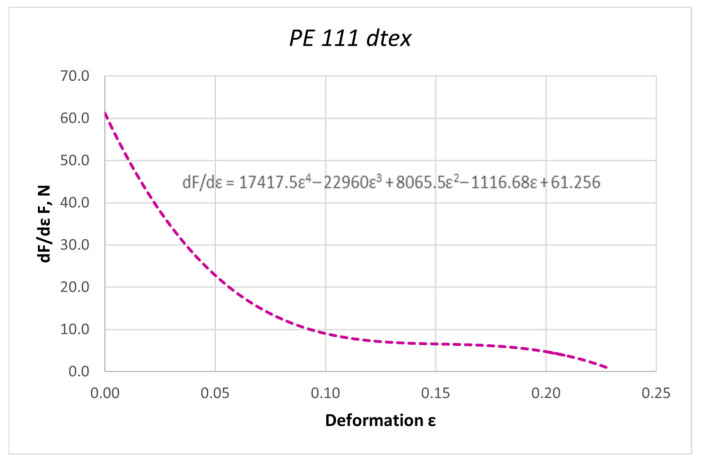
The course of the first derivative of the approximated characteristic F = f(ε) for the PES 111 dtex polyester thread (nonlinear modulus of elasticity).

The obtained results of approximation and the first derivative are presented as an example for the PES 111 dtex polyester thread in Figure 5 and Figure 6.

#### 3.2.3. Determination of the Nonlinear Viscosity Coefficient by Measuring the Forces During Stress Relaxation in the Threads

**Figure 7:**
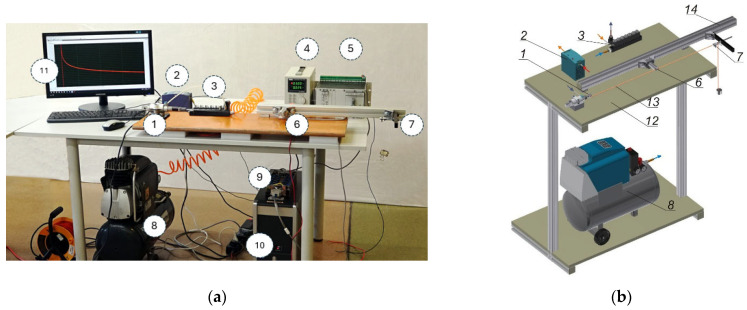
The device for measuring forces during stress relaxation in threads, (**a**) photograph, (**b**) diagram: 1—moveable clamp (pneumatic actuator-piston); 2—power supply unit DC24V; 3—solenoid valve; 4—tensometer bridge power supply; 5—power supply for the signal amplifier; 6—strain gauge sensor; 7—stationary clamp; 8—compressor; 9—sensor signal amplifier; 10—computer with an analog–digital transmission measurement card; 11—monitor; 12—base; 13—thread; 14—runner.

A device was designed and built to determine the viscosity coefficient η by measuring the forces during stress relaxation in the threads (Figure 7).

The tested thread was clamped between a movable clamp (1) and a stationary clamp (7) mounted on a runner (14) attached to the base (12). The distance between the clamps was constant and equal to 100 cm. The force values were measured using a strain gauge sensor (6) placed in the middle of the length of the stretched thread section. The movable clamp was a pneumatic actuator-piston with a maximum stroke of 1 inch. Air from the compressor (8) was supplied to the solenoid valve (3) at a pressure of 5 bar. At the time of measurement, a voltage of 24 V DC was switched on to the solenoid valve coil to open the air flow from the compressor to the actuator, causing the piston to move by a constant value Δl.

The measurement was started by securing the thread (13) in the movable clamp (1); after passing the thread through the stationary clamp (7), it was subjected to a preload P0 (the pretension value of 0.5 cN/tex was assumed for each of the threads). After placing the thread in the strain gauge sensor (6), the stationary clamp was blocked. Then, by moving the piston of the pneumatic actuator, the movable clamp was moved by a constant value Δl = 20 mm. The signal from the sensor, after amplification in the tensometric amplifier, was processed by the data acquisition card. The measured changes in the thread tension were recorded on the computer in the form of graphs U = f(t), where U—signal voltage in V, t—time in s. The Data Logger Application1—Advantech program [26,27] was used to record the results. Since the measuring system recorded the values of electric voltage in volts, it was necessary to calibrate the measuring sensor to convert them into force values in newtons. The obtained calibration curve was approximated by a linear function.

**Figure 8:**
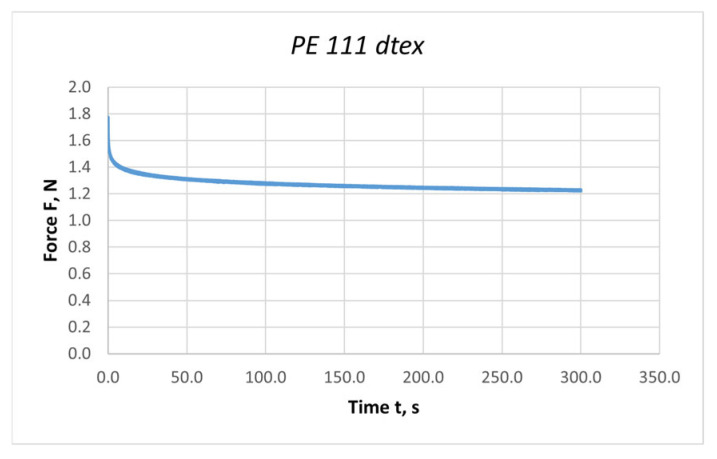
Graph of the average value of forces during stress relaxation in threads subjected to the stretching process: PES 111 dtex polyester thread; length of the stretched section l = 1000 mm; elongation Δl = 20 mm.

The length of the stretched thread section was 1000 mm, and the measurement time was 300 s. The Microsoft Excel computer program was used to process the measurement results. An example graph of the force values during stress relaxation for a polyester thread with a linear density of 111 dtex is shown in Figure 8.

The viscosity coefficient was calculated based on the Formula (15) [28] by substituting the values of individual quantities obtained during the stress relaxation process in the threads subjected to the stretching process. During the analysis of the results, it was observed that after 4 min, the changes in the force values were minimal; therefore, the relaxation time of 240 s was assumed for calculating the viscosity coefficient.

(15) $$\eta =\frac{{-t}_{\left(i\right)}\cdot c_{1}}{\ln\left(\frac{F_{(i)-c\cdot \varepsilon }}{c_{1}\cdot \varepsilon }\right)}$$

where η—viscosity coefficient; ε—relative elongation; c, c_1_—relative spring stiffness values; F_(i)_—force; t_(i)_—time.

The values of the relative spring stiffness c and c1 were calculated from the relationships presented below. The value of the relative extension was constant and equal to ε = 0.02. The values of the maximum and minimum forces were read from the relaxation curves.

(16) $$c=\frac{F_{min}}{\varepsilon }$$

(17) $$c_{1}=\frac{F_{max}}{\varepsilon }-c$$

where *F_min_*—minimum force value read from the relaxation curve for 240 s; *F_max_*—maximum force value.

**Figure 9:**
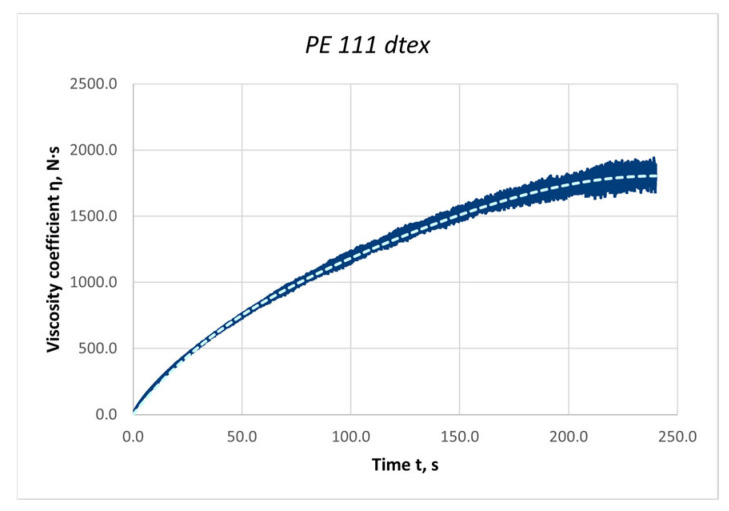
Graph of the non-linear viscosity coefficient value determined for the PES 111 dtex polyester thread.

An example graph of the viscosity coefficient value obtained for the PES 111 dtex polyester yarn is shown in Figure 9.

As a result of the approximation of the viscosity coefficient curves for individual threads, equations were obtained, which were then used to calculate the nonlinear viscosity coefficient for each variant, taking into account the dynamic stretching time of the thread.

### 3.3. Verification of Selected Rheological Models in the Autodesk^®^ Inventor^®^ Environment

The numerical simulation of the dynamic thread stretching phenomenon was performed in the Autodesk^®^ Inventor^®^ Professional 2023 software environment [29]. The Dynamic Simulation Module (MSD) of this program, allows solving ordinary differential equations using the Runge–Kutta convergence method or the Gaussian elimination method [30,31]. The program contains two types of elements: parts and joints. Parts are defined as a generalized mass with a center of gravity defining the position of the part in space. Joints are massless and define the nature of the mutual interaction of parts.

**Figure 10:**
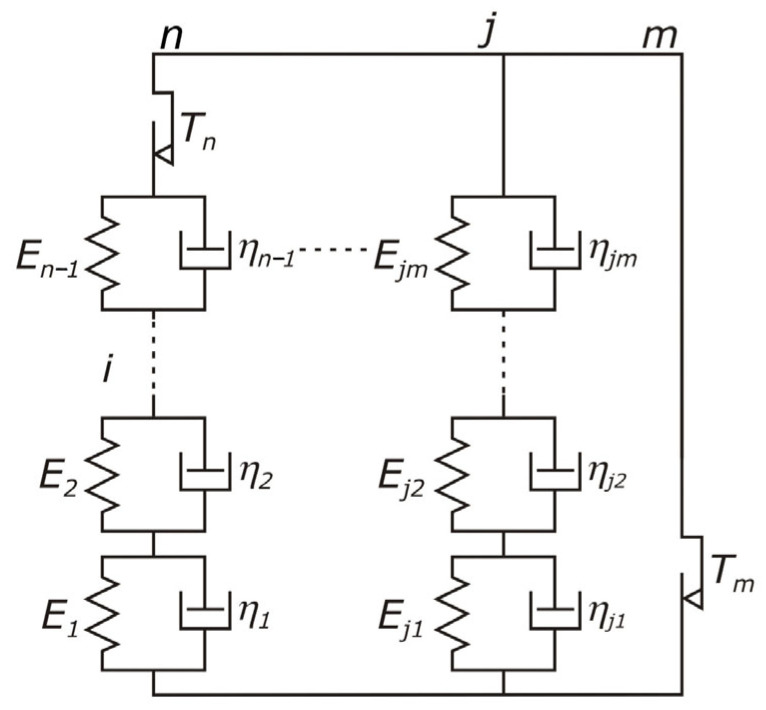
Generalized rheological model of the thread.

The Dynamic Simulation Module (DSM) enables the construction of rheological models characterized by a set of parameters describing elastic and damping properties and friction. They can be defined as constant or variable values related to time, displacement, velocity, and acceleration. This allows the analysis of dynamic systems with the inclusion of nonlinear parameters. In the theoretical research methodology, a generalized rheological model (Figure 10) consisting of an unlimited number of elasticity, viscosity, and friction terms connected in series and parallel was used to analyze the dynamic stretching processes of threads and textiles.

**Figure 11:**
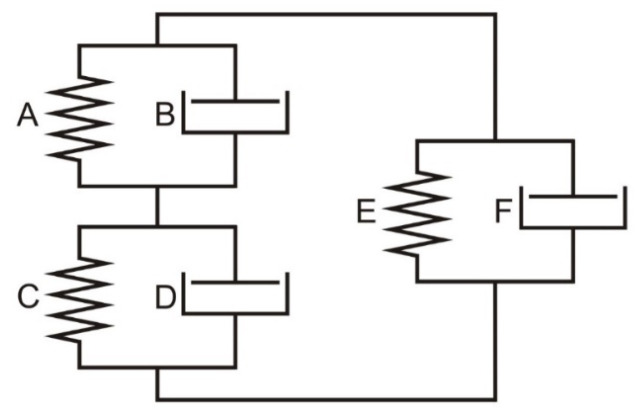
A system of connected Kelvin–Voigt models built in the Inventor program environment.

In accordance with the adopted assumptions for the description of mechanical properties of threads subjected to dynamic stretching using nonlinear three-parameter models, the procedures for building such models in the Autodesk^®^ Inventor^®^ software environment were adopted. A special virtual dynamic model (Figure 11) was built from three appropriately connected Kelvin–Voigt rheological models. It allows for a simple way to obtain each of the models selected for the analysis of the dynamic stretching process of threads by substituting zero values of the coefficients of unused elements.

To verify the correctness of the virtual model, a simulation of the stretching process of polyester threads was carried out based on two two-parameter rheological models, i.e., the Maxwell model and the Kelvin–Voigt model. The simulation required defining the constant parameters of the models and defining their elastic and viscous properties, such as the static elasticity coefficient E_K_, the elasticity coefficient determined for dynamic thread loads E_dM_, and the viscosity coefficient η. The values of the individual parameters were determined based on the results of laboratory tests presented in the articles [32,33].

As a result of the numerical simulation, the force characteristics as a function of time F = f(t) were obtained. For the same parameter values adopted for model verification, the state equations of the Kelvin–Voigt model and the Maxwell model were solved analytically using an Excel (Microsoft 365) spreadsheet. The dynamic response of the system to the given, time-varying extension was obtained in the form of a force diagram.

To verify the correctness of the numerical simulation, the obtained characteristics ofanalytical and model calculations were compared. The obtained curves coincide, which indicates the high accuracy of the simulations performed. The agreement of the numerical simulation results with the analytical solutions of the equations of state of simple rheological models provides a basis for assuming the correctness of the simulation results for more complex models.

### 3.4. Modelling the Dynamic Thread Stretching Process for Nonlinear Rheological Models

**Figure 12:**
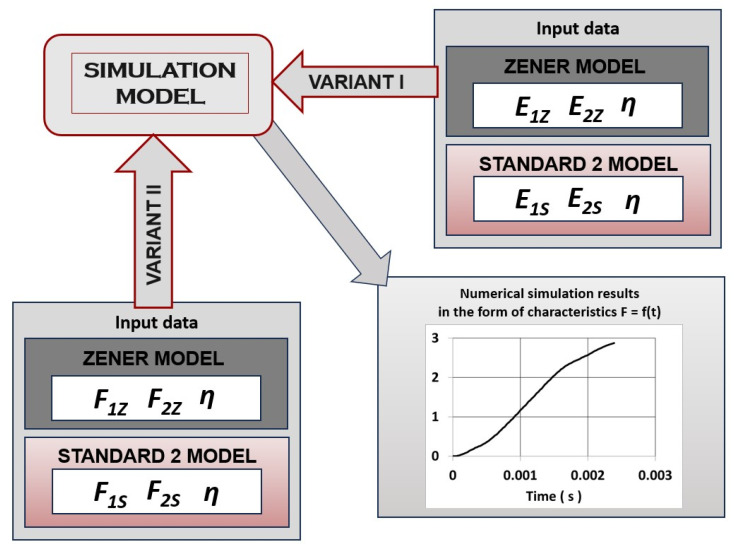
Simulation algorithm of the dynamic thread stretching process using the Zener and Standard 2 rheological models in Inventor^®^ for two input data input variants. E_1Z_, E_2Z_—elasticity coefficient for the Standard Model 1 (Zener), E_1Z_ = f(ε), E_2Z_ = f(ε); E_1S_, E_2S_—elasticity coefficient for Standard Model 2, E_1S_ = f(ε), E_2S_ = f(ε), η—viscosity coefficient values, η = f(t); F_1Z_, F_2Z_—values of elasticity coefficients for the Zener model in the form of force characteristics as a function of elongation, F_1Z_, F_2Z_ = f(Δl); F_1S_, F_2S_—values of elasticity coefficients for the Standard 2 model in the form of force characteristics as a function of elongation, F_1S_, F_2S_ = f(Δl).

Determination of the static elasticity coefficient E_K_ and the dynamic elasticity coefficient Ed required determining the equations of the F = f(ε) curves obtained in the stretching process. As a result of the approximation, the individual characteristics were described by a fifth-degree polynomial. The approximation of the function resulted in approximation errors that influenced the change of the obtained values of the individual elasticity coefficients entered into the simulation model (Figure 12, VARIANT I). Therefore, to perform the numerical simulation of the dynamic thread stretching process, a model was used that allowed for the introduction of the elasticity coefficient values directly in the form of force characteristics as a function of elongation F = f(Δl) (Figure 12, VARIANT II). In both variants, the viscosity coefficient values were declared in the form of a function η = f(t), and the response of the system was the force characteristics as a function of time F = f(t).

The Zener model was obtained by substituting zero values of the coefficients in the elements B, C, and F (Figure 11).

**Figure 13:**
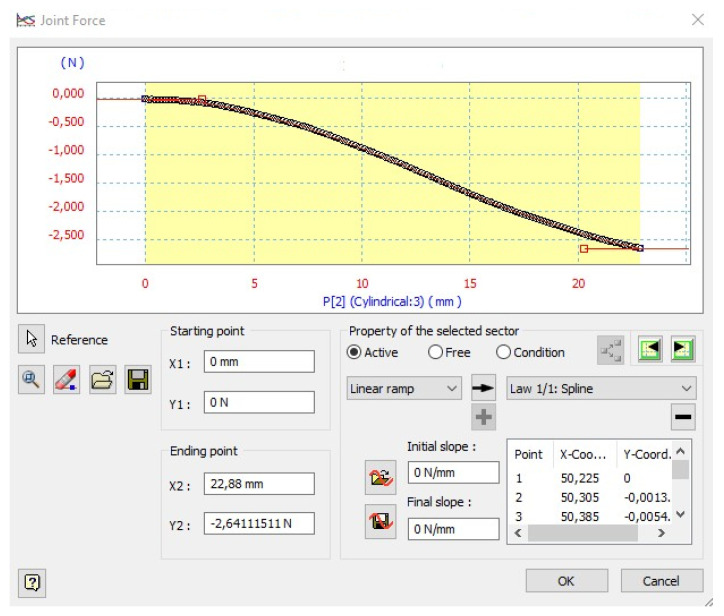
View of the dialog window with the force diagram F = f(Δl) obtained during dynamic stretching; data for the PES111-20 ms-200 mm variant.

To simulate the dynamic process of thread stretching, the values of individual parameters were assigned to the appropriate elements of the rheological models. The ability to define the range of parameter values, in this case, the force and viscosity coefficient as well as their characteristics as functions of time or deformation, is provided by a dialogue window. An example view of such a window is shown in Figure 13.

**Figure 14:**
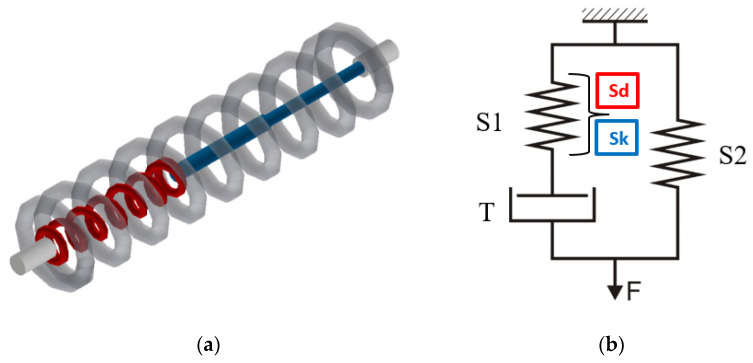
Zener model created in Inventor^®^: (**a**) visualization (grey-spring S2, red-spring S1, blue-dashpot T); (**b**) diagram.

In the Zener model (Figure 14b), according to the relationship (7), the S1 element was a combination of two modules Sd and Sk, taking into account the difference in the force value in the elongation function F = f(Δl) obtained during dynamic and static stretching of the threads. In the dialog window marked with the symbol S2, the values of the force in the elongation function obtained as a result of static excitation were loaded. The values of the viscosity coefficient related to the length of the stretched yarn section were loaded in the dialog window of the T element.

Creating the Standard Model 2 required setting the coefficients in the following elements of the system to zero: B, E, and F (Figure 11).

**Figure 15:**
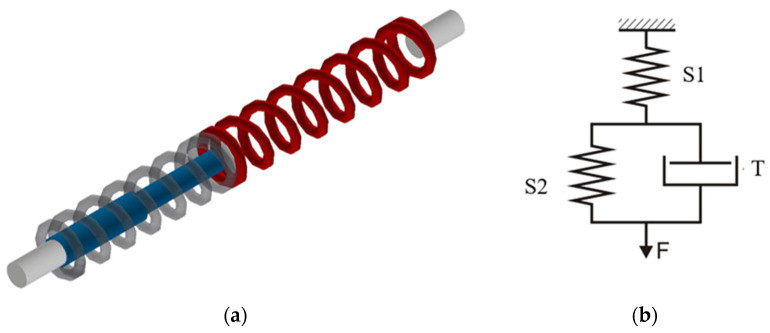
Standard Model 2 created in Inventor^®^: (**a**) visualization (grey-spring S2, red-spring S1, blue-dashpot T); (**b**) diagram.

In the Standard Model 2 (Figure 15b), in the tab defining the member marked by S1, the values of force in the function of elongation F = f(Δl) obtained during dynamic stretching of the threads were entered. In the dialog window marked by the symbol S2, the values of force in the function of elongation obtained during static stretching were loaded. The program application used for modeling takes into account the relationships between the coefficients of elasticity (14). The values of the viscosity coefficient related to the length of the stretched thread section were loaded in the dialog window of the member T.

Additionally, in the part of the model representing the Kelvin–Voigt model, the stretching speed value was declared. For individual variants, the actual stretching times of the threads were also declared.

## 4. Model Verification by Comparative Analysis of Real and Model Characteristics of the Dynamic Thread Stretching Process

The comparative analysis of the real and model characteristics was performed in the Excel environment.

Due to the extensive research material, Figure 16, Figure 17, Figure 18, Figure 19, Figure 20, Figure 21, Figure 22, Figure 23, Figure 24 and Figure 25 present exemplary characteristics F = f(t) obtained as a result of the comparative analysis, where R_z_ denotes the values of the Pearson correlation coefficient obtained for the Zener model and R_S2_ for the Standard 2 model.

## 5. Analysis of the Results of Dynamic Thread Stretching Nonlinear Models Verification Tests

Comparison of characteristics obtained from experimental tests and numerical simulation of nonlinear models was considered depending on the stretching speed for five lengths of the stretched thread sections: l = 200, 400, 600, 800, and 1000 mm. As a result of the comparative analysis of experimental and numerical simulation characteristics, it can be seen that the Standard 2 model better reflects the nature of the course of the experimental curves for all analyzed cases. A better fit of the model characteristics to the actual curves was obtained for polyamide threads. In the case of polyester threads, the degree of fit decreases slightly with the increase of the linear mass of the thread. Out of 150 analyzed variants, the model curves coincide with the experimental characteristics for the Zener model in 80 cases and for the Standard 2 model in 34 variants. In most cases, both model characteristics coincide with the actual ones in the range of initial values of the stretching time, which increases with the increase of the stretching speed.

The slight discrepancies of the model curves and experimental characteristics result from the universal algorithm of the simulation program, in which the accuracy of the dynamic analysis of ultralight bodies is limited. Errors in numerical models may arise as a result of input data errors, which may be subject to measurement errors, rounding errors resulting from the limited number of digits in the numerical representation, or calculation errors that approximate solutions of mathematical equations. The accuracy of the theoretical model built in Autodesk Inventor depends, among other things, on the accuracy of determining the nonlinear coefficients of elasticity and viscosity. These coefficients were determined using experimental methods, which may be subject to systematic errors resulting from the measurement of force during the dynamic thread stretching process.

The verification of the degree of matching of the obtained model and experimental characteristics was carried out using the Pearson correlation coefficient analysis [34,35]. The lowest value of the correlation coefficient R = 0.9940 was noted when comparing the actual and model characteristics obtained for the Zener model and the PES 167 dtex polyester thread, while the highest R = 0.9998 in the case of both models for the PA 56 dtex polyamide thread. The average values of the correlation coefficient R = 0.9986 indicate high reliability of the adopted assumptions of nonlinear rheological models and the procedures for solving them in determining the dynamic properties of linear textile materials

## 6. Conclusions

1. The conducted literature study shows a large variety of tested textile materials (yarns, threads, and flat textiles) and rheological models describing the behavior of textiles in the process of tensile excitations. The identification of mechanical properties of textile processes and textile materials was performed using rheological models of viscoelastic bodies, including multi-parameter, three-element linear, and non-linear models, and taking into account friction elements;
2. A generalized rheological model of a thread was developed, which consists of a series and parallel connection of an unlimited number of elasticity, damping, and friction elements;
3. Simulation studies of the dynamic thread stretching process were based on two three-parameter rheological models, i.e., the Zener model and the Standard Model 2. A virtual analogue model was built in the Autodesk^®^ Inventor^®^ software environment based on appropriately connected modules of Kelvin–Voigt rheological models. The correctness of the adopted assumptions and the construction of the software algorithm for solving the models were verified. Procedures for determining nonlinear coefficients of elasticity and viscosity of three-parameter rheological models were defined;
4. For these models, calculations were performed using the developed tensile process simulation algorithm, which resulted in force as a function of time characteristics;
5. The results of the verification of the degree of matching of the characteristics obtained based on the comparative analysis of the experimental and model tests of the dynamic thread stretching process showed a very strong correlation for the average value of the Pearson correlation coefficient R = 0.9986, which leads to the conclusion about the usefulness of the used tools for the physical description of the thread (textile) stretching process in the form of nonlinear rheological models;
6. The presented research proves the possibility of using numerical modeling to describe the physical phenomena of the dynamic stretching process of threads (textiles) using multi-parameter non-linear rheological models.

## Figures and Tables

**Figure 16:**
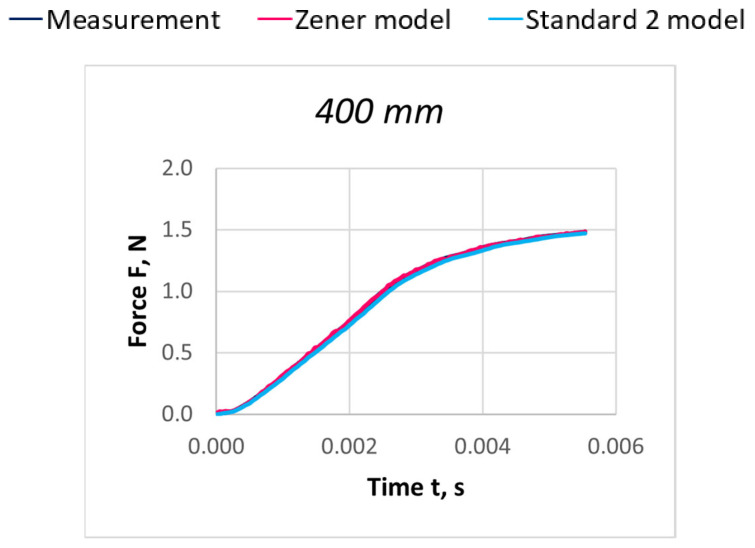
Comparison of real and model characteristics for the PA 56 dtex polyamide thread obtained for the stretching speed of 20 m/s and the stretched section length l = 400 mm; R_Z_ = 1.0000; R_S2_ = 0.9998.

**Figure 17:**
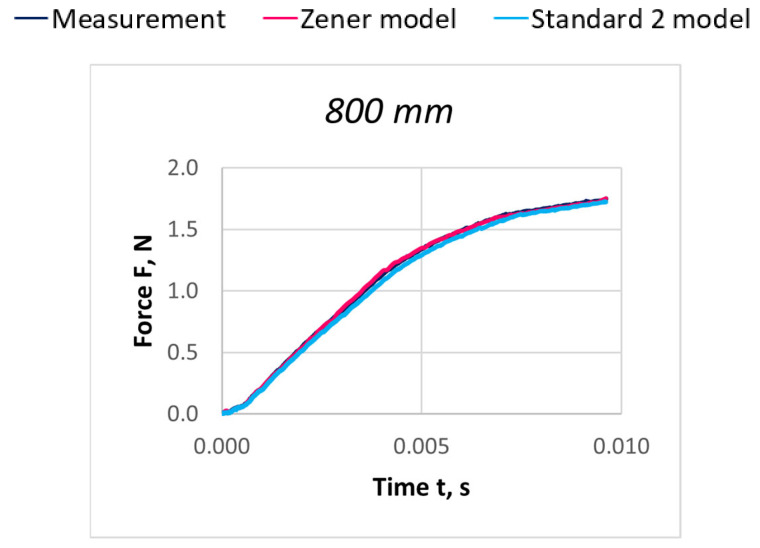
Comparison of real and model characteristics for the PA 56 dtex polyamide thread obtained for the stretching speed of 20 m/s and the stretched section length l = 800 mm; R_Z_ = 0.9998; R_S2_ = 0.9997.

**Figure 18:**
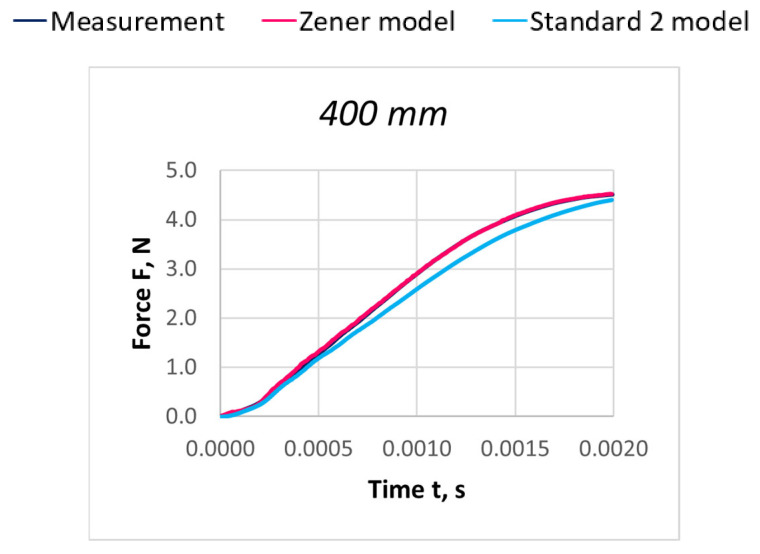
Comparison of real and model characteristics for the PA 156 dtex polyamide thread obtained for the stretching speed of 40 m/s and the stretched section length l = 400 mm; R_Z_ = 0.9999; R_S2_ = 0.9987.

**Figure 19:**
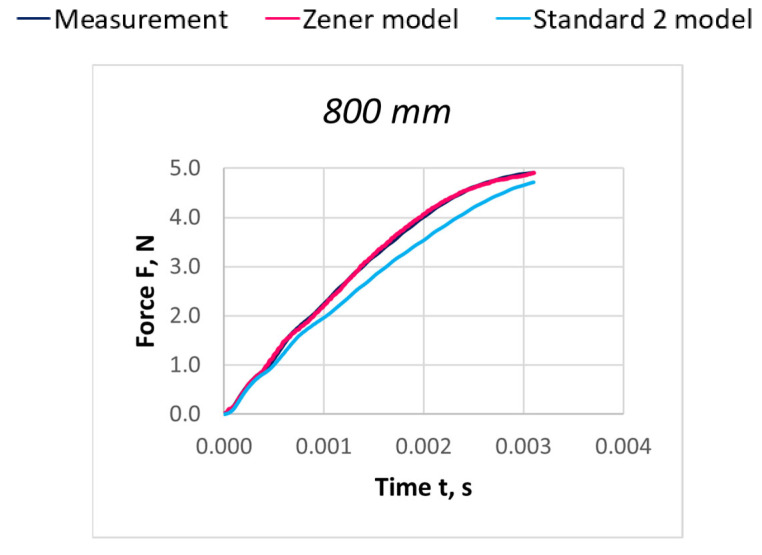
Comparison of real and model characteristics for the PA 156 dtex polyamide thread obtained for a stretching speed of 40 m/s and a stretched section length of l = 800 mm; R_Z_ = 0.9998; R_S2_ = 0.9978.

**Figure 20:**
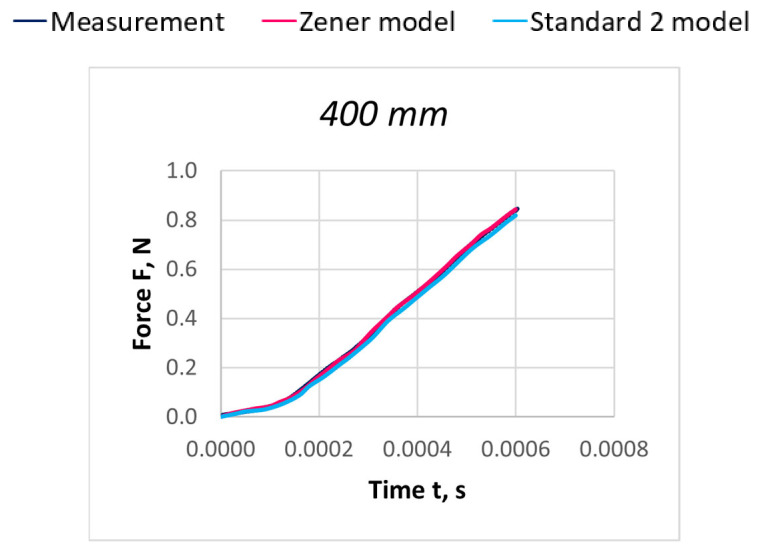
Comparison of real and model characteristics for the PES 55 dtex polyester thread obtained for a stretching speed of 60 m/s and a stretched section length of l = 400 mm; R_Z_ = 0.9999; R_S2_ = 0.9999.

**Figure 21:**
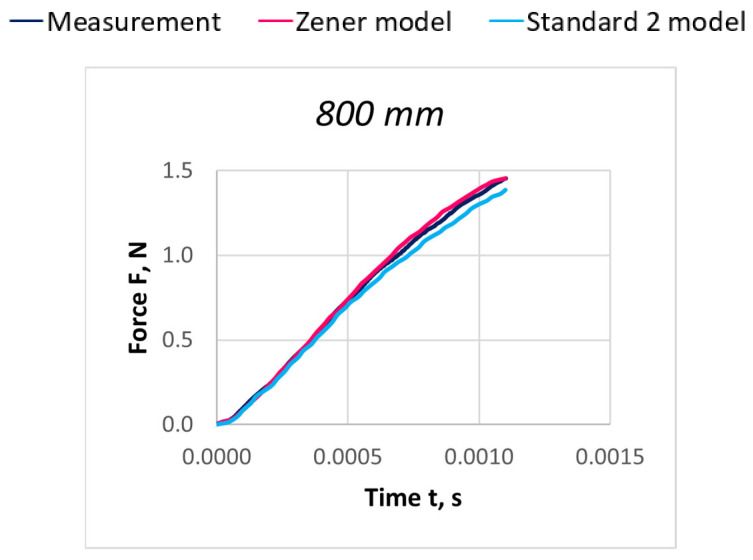
Comparison of real and model characteristics for the PES 55 dtex polyester thread obtained for a stretching speed of 60 m/s and a stretched section length of l = 800 mm; R_Z_ = 0.9998; R_S2_ = 0.9999.

**Figure 22:**
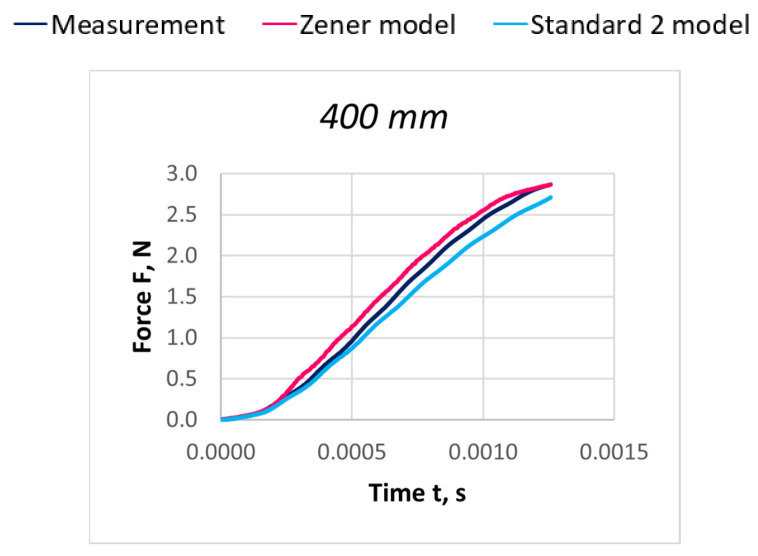
Comparison of real and model characteristics for the PES 111 dtex polyester thread obtained for a stretching speed of 30 m/s and a stretched section length of l = 400 mm; R_Z_ = 0.9979; R_S2_ = 0.9998.

**Figure 23:**
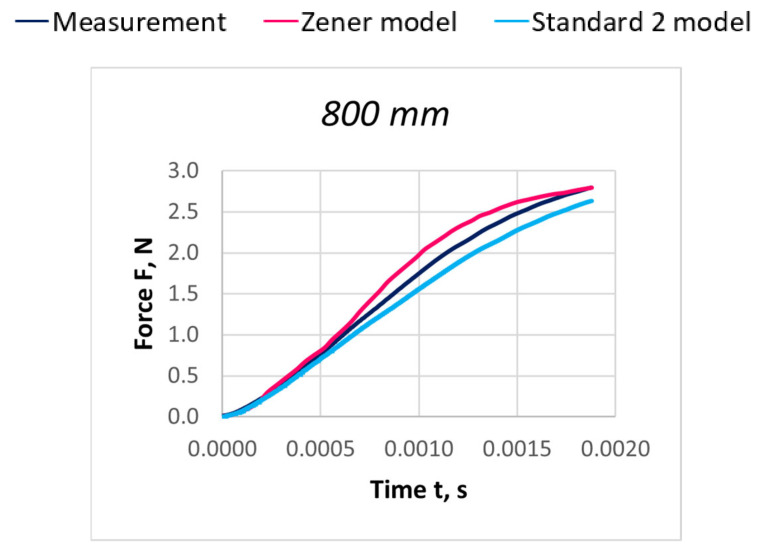
Comparison of real and model characteristics for the PES 111 dtex polyester thread obtained for a stretching speed of 30 m/s and a stretched section length of l = 800 mm; R_Z_ = 0.9969; R_S2_ = 0.9995.

**Figure 24:**
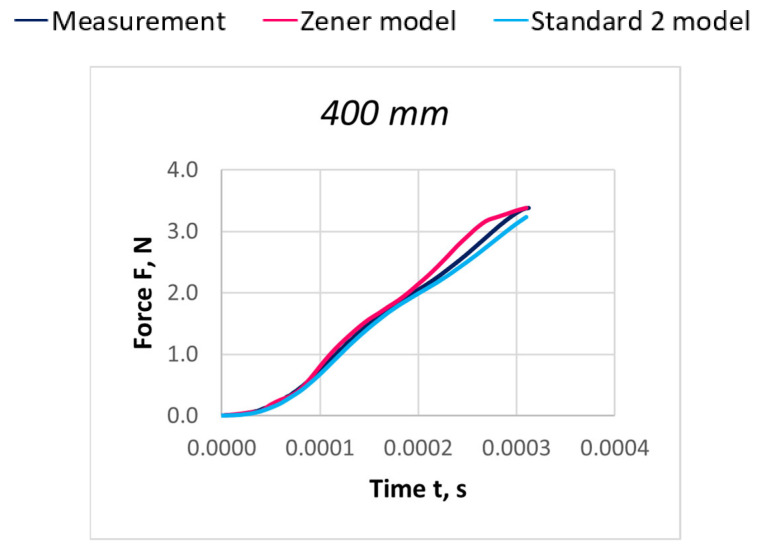
Comparison of real and model characteristics for the PES 167 dtex polyester thread obtained for a stretching speed of 100 m/s and a stretched section length of l = 400 mm; R_Z_ = 0.9985; R_S2_ = 0.9997.

**Figure 25:**
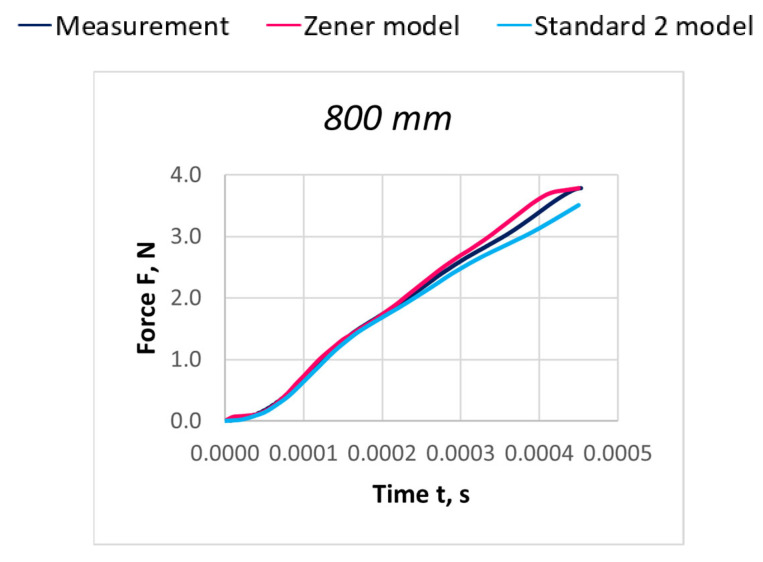
Comparison of real and model characteristics for the PES 167 dtex polyester thread obtained for a stretching speed of 100 m/s and a stretched section length of l = 800 mm; R_Z_ = 0.9993; R_S2_ = 0.9993.

## Data Availability

The original contributions presented in this study are included in this article, and further inquiries can be directed to the corresponding author.
